# Successful Pregnancy Outcome in a Patient With Crohn’s Disease and Prior Intestinal Surgery: A Case Report

**DOI:** 10.7759/cureus.106444

**Published:** 2026-04-04

**Authors:** Emmanouil M Xydias, Vasiliki Koutsia, Georgios Toulios, Zoe Kotoula, Ioannis Tsartsalis, Konstantinos Zachos, Aikaterini Kariotaki, Iliana Kotoula, Apostolos C Ziogas, Ioannis Thanasas

**Affiliations:** 1 Department of Obstetrics and Gynecology, EmbryoClinic IVF, Thessaloniki, GRC; 2 Department of Obstetrics and Gynecology, General Hospital of Trikala, Trikala, GRC; 3 Department of Surgery, General Hospital of Trikala, Trikala, GRC; 4 Department of General Surgery, General Hospital of Trikala, Trikala, GRC; 5 Department of Anesthesiology, General Hospital of Trikala, Trikala, GRC; 6 Department of Obstetrics and Gynecology, University of Thessaly, Larissa, GRC

**Keywords:** case report, complications, crohn’s disease, diagnostic approach, enterectomy, idiopathic inflammatory bowel diseases, pharmacological management, pregnancy

## Abstract

Crohn’s disease (CD) is a chronic autoimmune disorder of the GI system that primarily affects young individuals of reproductive age and is often associated with pregnancy. This case report describes the successful outcome of a pregnancy in a 32-year-old primigravida woman with CD, who had undergone enterectomy five years prior. At the time of conception and throughout the entire pregnancy, the disease was in remission under pharmacological treatment. The pregnancy, under systematic monitoring, progressed smoothly for the mother, fetus, and neonate. After completion of the 38th week of gestation, an elective cesarean section was performed without complications. The puerperium was uneventful. Eight months later, the disease remained in complete remission. Following the case description, a brief review of the literature on CD in pregnancy is provided.

## Introduction

Inflammatory bowel disease (IBD) is a chronic intestinal inflammation that may present with a spectrum of local intestinal manifestations and extraintestinal complications. A characteristic feature of this condition is the combination of innate and adaptive immune dysfunction, which, in association with environmental factors, may trigger the onset and perpetuation of inflammation [1]. The exact etiology of IBD remains unknown. Genetic predisposition, an altered intestinal microbiome, and environmental factors that have not yet been clearly defined are considered to contribute to the pathogenesis of the disease [2]. Among IBDs, ulcerative colitis and Crohn’s disease (CD) are most commonly described [3].

CD is characterized by segmental, transmural inflammatory lesions that may occur in any part of the GI tract. The disease most commonly involves the terminal ileum, the ileocecal region, the colon, and the perianal area [4]. CD appears to affect women more frequently than men and usually involves individuals between 10 and 40 years of age, with the highest incidence observed between 15 and 20 years. In 2004, the incidence of CD was estimated at 5.6 cases per 100,000 of the general population and is significantly higher in the United States and European countries compared to Asia and Africa [5,6]. CD is often associated with pregnancy. It is estimated that approximately 25% of patients with CD have their first pregnancy after the diagnosis of the disease. Although CD is prevalent in women of reproductive age, pregnancy itself does not increase the incidence of CD [7].

Herein, we describe the case of a pregnant woman with CD who had undergone intestinal surgery five years earlier and whose pregnancy and delivery progressed without complications. The management of CD in pregnant women, based on the effects of pregnancy on the disease, the effects of the disease on pregnancy, and the timing and mode of delivery in order to achieve optimal outcomes for the mother and fetus/neonate, constitutes the main focus of the discussion in the present study.

## Case presentation

The case concerns a 32-year-old primigravida woman who presented to the outpatient clinic of the Department of Obstetrics and Gynecology of the General Hospital of Trikala, Greece, reporting six weeks of secondary amenorrhea. A urine pregnancy test was positive, and transvaginal ultrasonography confirmed a normally progressing intrauterine pregnancy.

From the medical history, it was established that the patient had CD localized to the ileocecal region, predominantly in the initial segment of the ascending colon, which had first been diagnosed ten years earlier. Five years prior, due to resistance to pharmacological treatment and severe exacerbations, the disease had been managed surgically with resection of the affected intestinal segment (ileocolectomy) and anastomosis of the healthy ends. Prior to conception, the disease was in remission under treatment with sulfasalazine. The patient had no other comorbidities. She reported no previous pregnancies, miscarriages, or history of infertility. The current pregnancy resulted from spontaneous conception. Her body weight was within normal limits (BMI = 22.4 kg/m²).

Monitoring of the patient until the 10th week of pregnancy was uneventful. Nausea and occasional vomiting were managed with dietary recommendations and oral antiemetic medications. Hospital admission and intravenous administration of fluids, electrolytes, or antiemetic drugs were not required. Hemoglobin levels, inflammatory markers, coagulation parameters, and biochemical tests remained within normal limits throughout the pregnancy (Table 1).

**Table 1:** Laboratory tests of the patient throughout pregnancy APTT, activated partial thromboplastin time; Cr, creatinine; Hb, hemoglobin; Ht, hematocrit; INR, international normalized ratio; NEUT, neutrophils; SGOT, serum glutamic oxaloacetic transaminase; SGPT, serum glutamic pyruvate transaminase; TBIL, total bilirubin

Laboratory tests	First trimester	Second trimester	Third trimester	Normal laboratory values
Ht	38.10%	36.30%	35.70%	37.7-49.7%
Hb	12.7 g/dL	12.1 g/dL	11.6 g/dL	11.8-17.8 g/dL
WBC	7.9 × 10³/µL	10.1 × 10³/µL	10.7 × 10³/µL	4-10.8 × 10³/µL
NEUT	67%	71%	73%	40-75%
CRP	0.3 mg/dL	0.4 mg/dL	0.3 mg/dL	0.5 mg/dL
APTT	31.6 seconds	29.9 seconds	30.1 seconds	24.0-35.0 seconds
INR	0.95	0.91	0.99	0.8-1.2
Cr	0.45 mg/dL	0.55 mg/dL	0.55 mg/dL	0.40-1.10 mg/dL
K⁺	4.3 mmol/L	3.9 mmol/L	4.1 mmol/L	3.5-5.1 mmol/L
Na⁺	139.8 mmol/L	140.1 mmol/L	139.9 mmol/L	136-145 mmol/L
TBIL	0.7 mg/dL	0.8 mg/dL	0.6 mg/dL	0-1.2 mg/dL
SGOT	25 IU/L	27 IU/L	21 IU/L	5-33 IU/L
SGPT	31 IU/L	36 IU/L	29 IU/L	10-37 IU/L

The glucose tolerance curve was normal. Noninvasive prenatal screening during the first trimester, including measurement of nuchal translucency in combination with the pregnancy-associated plasma protein A test, and during the second trimester, including a detailed second-level ultrasound examination, revealed no abnormal findings. Throughout the pregnancy, iron and folic acid supplements were administered at a dose of 5 mg daily. In consultation with the treating gastroenterologist, to avoid exacerbations of the disease, continuation of oral pharmacological treatment with sulfasalazine at a dose of 2 g per 24 hours was recommended.

**Figure 1:**
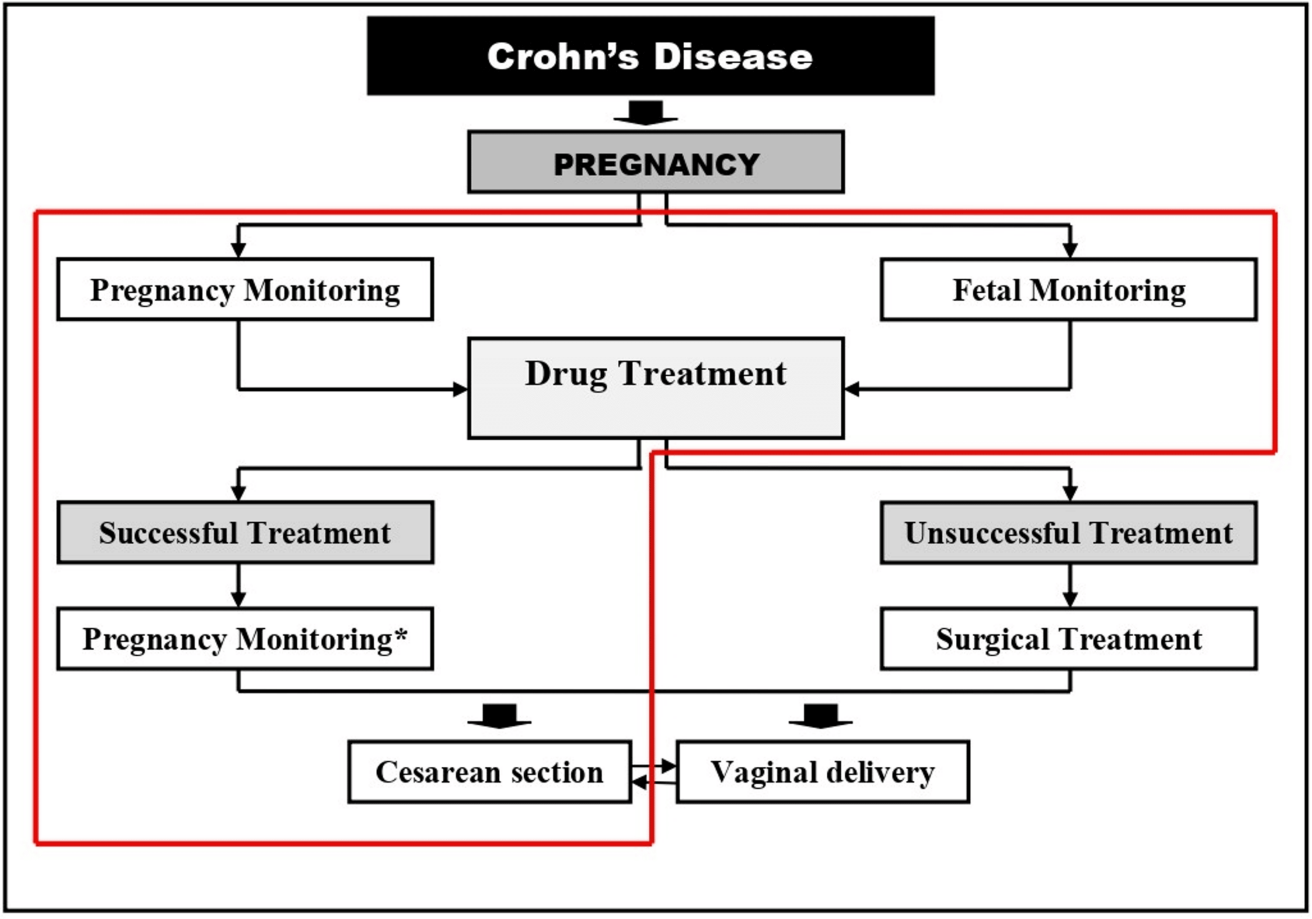
Flowchart of the CD management protocol during pregnancy in our clinic Steps followed in the management of the present case are highlighted with a red outline. ^*^ Monitoring of the pregnant woman with continuation of drug treatment CD, Crohn’s disease

During the third trimester, the patient’s course under systematic monitoring was uneventful. Ultrasonographic assessment of fetal growth, sonographic evaluation of amniotic fluid volume, and Doppler ultrasonography of blood flow in the umbilical vessels were all within normal limits. In addition, resting cardiotocographic monitoring, performed weekly from the 32nd week of gestation until delivery, was normal. After completion of the 38th week of pregnancy, the patient delivered a female neonate weighing 2,980 g by elective cesarean section (Figure 1).

Following an uneventful postoperative course, the puerperal woman and the neonate were discharged from the clinic on the fourth postoperative day. The puerperium remained uneventful, with no further complications for either the mother or the neonate. Eight months later, the woman, under treatment with sulfasalazine, remained in complete remission, with no clinical or laboratory findings indicative of disease.

## Discussion

Diagnosis of CD in pregnant women is not straightforward. Concerns regarding the safety of endoscopic evaluation during pregnancy, as well as the difficulty in interpreting various hematological parameters in pregnant women, complicate the laboratory, imaging, and histological confirmation of the clinical diagnosis [8]. Although endoscopy-related adverse outcomes for the mother and the fetus/neonate do not appear to be increased in any of the three trimesters of pregnancy, the available data regarding endoscopic procedures in pregnant women with ulcerative colitis and CD are limited. It is considered that lower GI endoscopy in pregnant women with IBD is indicated only in cases of absolute and severe clinical necessity [9].

The typical clinical manifestations of CD include chronic diarrheal bowel movements, with or without the presence of blood or mucus, which constitute the main clinical feature. In addition, abdominal pain, fever, and weight loss are among the most common clinical manifestations of the disease [10]. In other cases, symptoms from the perianal region, such as fistulas, abscesses, and fissures, are more frequently observed. Furthermore, extraintestinal manifestations of the disease include peripheral arthritis, ankylosing spondylitis, skin lesions such as erythema nodosum and pyoderma gangrenosum, ocular involvement (conjunctivitis, episcleritis, or iriditis), oral lesions (aphthous ulcers), and primary sclerosing cholangitis [11].

Moreover, vitamin K deficiency associated with short bowel syndrome following enterectomy represents a rare postoperative complication of CD, which during pregnancy may lead to severe hemorrhagic complications in the mother and the fetus/neonate [12]. In our patient, no clinical symptoms related to CD were observed throughout the entire duration of pregnancy. Diarrheal bowel movements, with or without blood or mucus, abdominal pain, fever, or extraintestinal manifestations of the disease were not observed in any trimester of pregnancy. Additionally, hemoglobin levels, white blood cell count, neutrophil percentage, C-reactive protein, coagulation parameters, and biochemical tests remained within normal limits during pregnancy (Table 1).

The course of IBDs in pregnant women primarily depends on disease activity at the time of conception, during pregnancy, and in the postpartum period. Hormonal and immune system changes that normally occur during pregnancy are estimated to influence the activity of IBDs [13]. Disease control prior to conception and throughout pregnancy constitutes the cornerstone for the successful management of pregnant women with IBD [14].

Earlier studies have shown that in approximately two-thirds of patients with active disease at the time of conception, the disease will remain active or worsen during pregnancy, whereas in the remaining one-third of patients, remission is expected during pregnancy, particularly in the first trimester. Conversely, if the disease is in remission at the time of conception, about two-thirds of pregnant women are not expected to experience disease exacerbation, while in the remaining one-third of cases, relapses may occur during pregnancy and the puerperium. If the disease manifests for the first time during pregnancy, its course is generally expected to be unfavorable [15,16]. More recently, it has been reported that the risk of CD relapse during pregnancy, when the disease is in remission at conception, is 20-25%, a rate approximately similar to that of nonpregnant women. However, if the disease is active at conception, the risk of relapse doubles, reaching approximately 50% of cases [17,18]. In our patient, CD had been surgically treated five years prior to conception and remained in remission under pharmacological treatment with sulfasalazine at the time of conception and throughout the entire pregnancy. This is considered one of the factors contributing to the smooth progression of pregnancy without complications.

CD, in turn, can negatively impact women who wish to achieve future pregnancy. This burden may affect the period before conception, during pregnancy, labor, and the lactation period. Bokemeyer et al., analyzing the results of their study, found that approximately one-quarter of patients (26.7%) with CD report sexual dysfunction, which can be particularly severe during disease flares [19]. Furthermore, fertility rates in patients with CD who have undergone intestinal resection are estimated to be lower compared to the general population [20]. In contrast, our patient, who had previous intestinal surgery, did not have difficulty conceiving.

In addition, limited knowledge regarding disease course during pregnancy and potential adverse effects of therapy on the mother and the fetus/neonate increases voluntary childlessness [21]. It is estimated that the rate of voluntary childlessness rises from 6.2% in the general population to approximately 18% in patients with CD [22]. CD often complicates pregnancy and may be associated with serious obstetric complications; however, remission for at least three months prior to conception and maintenance of therapy throughout pregnancy, as in our case, can lead to a favorable pregnancy outcome and significantly improve perinatal results [23]. Our case aligns with this observation. A recent study by Papathanasiou et al. demonstrated that the majority of Greek pregnant women with idiopathic IBD had favorable pregnancy outcomes, with the exception of those with active intestinal inflammation during gestation [24].

The risk of fetal loss depends on the presence or absence of active disease. Active disease during pregnancy can lead to adverse outcomes, such as preterm birth and low birth weight [25]. Additionally, higher rates of cesarean section are frequently observed in pregnant women with CD [26]. Finally, the literature has reported a unique case of rapidly progressing necrotizing perineal and perianal infection (Fournier’s gangrene) in a pregnant patient with CD [27].

Accurate initial assessment of CD and the potential complications that may arise for the mother and the fetus/neonate is of primary importance for guiding the most appropriate therapeutic decisions [28]. Pregnant women with CD, particularly when the disease is active, should be closely monitored by a specialized multidisciplinary team that includes a gastroenterologist, a surgeon, and an obstetrician-gynecologist. Furthermore, the establishment of dedicated clinical centers with trained physicians capable of providing specialized information on IBDs appears to be the optimal approach for managing disease flares in pregnant women [29].

In general, treatment of CD during pregnancy should follow the same guidelines as treatment in nonpregnant patients. Management of potential disease exacerbations during pregnancy is primarily pharmacological. Currently, several categories of medications, including aminosalicylates, corticosteroids, immunosuppressive agents, and antibiotics, play a role in the contemporary therapeutic approach to CD in pregnant women [21].

The most commonly used medications for the management of CD during pregnancy are aminosalicylates, primarily sulfasalazine and mesalamine. Sulfasalazine, administered in combination with folic acid supplements at a dose >2 mg/day, is considered the drug of choice for both active-phase treatment and maintenance therapy of CD. Analyzing the results of their meta-analysis, Rahimi et al. demonstrated that the use of aminosalicylates in pregnant women is not associated with an increased risk of spontaneous abortion, preterm birth, low neonatal birth weight, intrauterine death, or congenital neonatal malformations [30].

Corticosteroids, whose benefits are considered to outweigh their risks, should be administered at the lowest effective dose to control symptoms and prevent CD exacerbations. Although corticosteroids do not appear to be associated with an increased risk of congenital malformations in the fetus, their use for CD management during pregnancy carries an increased risk of gestational diabetes, low neonatal birth weight, preterm delivery, and infant infections within the first four months postpartum [31].

In cases where CD does not respond to maximum doses of aminosalicylates and corticosteroids, the use of immunosuppressive medications should be seriously considered. Azathioprine and mercaptopurine are currently regarded as safe during pregnancy, although potential risks, including teratogenicity, intrauterine growth restriction, and preterm birth, remain [32]. Methotrexate should be avoided both during pregnancy and in the three months prior to conception due to its high risk of teratogenicity [33]. The use of anti-tumor necrosis factor agents, such as infliximab (IFX) and adalimumab, does not appear to be associated with an increased risk of adverse pregnancy outcomes or congenital fetal anomalies [34]. Caution is advised in neonates born to mothers exposed to IFX, as they may be at increased risk of complications during the first year of life if administered live vaccines [35]. Additionally, studies have shown that the use of metronidazole and ciprofloxacin is not associated with adverse pregnancy outcomes or congenital malformations [36,37].

Finally, the information currently available regarding the safety of surgical intervention in pregnant women with CD is very limited. Although surgical management of CD is rarely required during pregnancy, it is generally recommended to be avoided due to the high risk of fetal loss associated with these procedures [38]. However, in pregnant women for whom surgical intervention is deemed necessary, it should be performed without delay by a multidisciplinary team, including gastroenterologists, colorectal surgeons, obstetricians, and neonatologists, to prevent more serious complications [39]. Generally, patients in this case undergo surgery prior to pregnancy, not during pregnancy.

The optimal timing and mode of delivery primarily depend on the presence or absence of complications related to CD. In cases of intrauterine fetal distress and/or severe maternal complications, induction of labor is required, with all the potential serious consequences associated with prematurity. In asymptomatic forms of the disease, and when there are no signs of fetal compromise, delivery is preferably carried out at term, as in our case. The choice of the most appropriate mode of delivery has been the subject of considerable debate in contemporary obstetric practice. According to many researchers, these women can have a vaginal delivery, while others advocate for elective cesarean section, as in our case. Most, however, agree that in patients with active perianal CD or a history of intestinal or perianal surgery, elective cesarean section appears to be preferable to vaginal delivery to avoid potential GI trauma [40,41].

## Conclusions

CD is an IBD that primarily affects young individuals and is often associated with pregnancy. As demonstrated by our case, patients with CD who have undergone previous intestinal surgery can conceive successfully and deliver without complications. Achieving pharmacological remission of the disease prior to conception and maintaining therapy throughout pregnancy is vital for the proper management of these patients. Management of pregnancy in women with CD can often be challenging and may require specialized care and a multidisciplinary approach. Caution is needed when generalizing these conclusions, and larger studies are required to validate these findings.
